# Heritability of clubfoot: a twin study

**DOI:** 10.1007/s11832-014-0562-7

**Published:** 2014-02-07

**Authors:** Vilhelm Engell, Jan Nielsen, Frank Damborg, Kirsten Ohm Kyvik, Karsten Thomsen, Niels Wisbech Pedersen, Mikkel Andersen, Søren Overgaard

**Affiliations:** 1 Department of Orthopaedic Surgery and Traumatology (Affiliated to Institute of Clinical Research, University of Southern Denmark, Denmark), Odense University Hospital, Sdr. Boulevard 29, 5000 Odense C., Denmark; 2 Odense University Hospital, Sdr. Boulevard 29, 5000 Odense C., Denmark; 3 Department of Orthopaedic Surgery, Lillebaelt Hospital, Kolding, Skovvangen 2-8, 6000 Kolding, Denmark; 4 Institute of Regional Health Research, University of Southern Denmark and Odense Patient data Explorative Network (OPEN), Odense University Hospital (Affiliated to: The Danish Twin Registry, Institute of Public Health, Denmark), Odense University Hospital, Sdr. Boulevard 29, 5000 Odense C., Denmark; 5 Spinesection, Hamlet Hospital, Copenhagen, Denmark; 6 Spinesection, Department of Orthopaedic Surgery, Lillebaelt Hospital, Middelfart, Østre Hougvej 55, 5500 Middelfart, Denmark

**Keywords:** Clubfoot, Twins, Heritability, Genetics, Aetiology

## Abstract

**Introduction:**

The aetiology of congenital clubfoot is unclear. Although studies on populations, families, and twins suggest a genetic component to the aetiology, other studies have identified environmental factors. The purpose of this study was to calculate heritability in order to determine to what extent genetic and/or environmental factors contribute to the aetiology of congenital clubfoot and to asses whether there was a change in the prevalence over time.

**Materials and Methods:**

The Odense based Danish Twin Registry is unique as it contains data on all the approximately 85,000 twin pairs born in Denmark over the last 140 years. All 46,418 twin individuals born from 1931 through 1982, who had earlier consented to contact, received a 17-page Omnibus questionnaire in the spring of 2002. Data were analysed with structural equation models to identify the best fitting aetiological model based on a balance of goodness-of-fit and parsimony and to estimate heritability.

**Results:**

We found an overall self-reported prevalence of congenital clubfoot of 0.0027 (95 % confidence interval 0.0022–0.0034). Fifty-five complete (both twins answered the question) twin pairs were identified representing 12 monozygotic, 22 same-sex dizygotic, 18 opposite-sex dizygotic, and 3 with unclassified zygosity. The model with only environmental factors (CE) was best fitting based on AIC, and the model with an additive genetic factor (ACE) came in second. Due to the small statistical power, we hypothesise that the model with both genetic and environmental effects (ACE) was the better model. Choosing the ACE-model we found a heritability of clubfoot of 30 %. Regression coefficient for age was −0.002 (−0.011 to 0.005), indicating that there has been no change in prevalence of clubfoot over the 50-year age span we examined.

**Discussion:**

We conclude that non-genetic factors must play a role, and a genetic factor might contribute, in the aetiology of congenital clubfoot.

## Introduction

The aetiology of congenital clubfoot is speculative and the influence of genetic and environmental factors remains unclear. Although studies on populations, families, and twins suggest a genetic component, the mode of inheritance does not fit the classic inheritance patterns [1–6].

Twin studies are useful when determining whether or not a disorder is genetic in aetiology. Monozygotic (MZ) twins inherit identical genes, while dizygotic (DZ) twins, like siblings, share 50 % of their genes. All twins share an identical environment before birth, and regarding congenital disorders, it is therefore possible to study the effect of genetic factors. Increased rates of double-affected MZ twins compared to DZ twins (concordance) indicate a genetic aetiology of a disorder. In clubfoot patients, the higher concordance in MZ twins (33 %) compared to DZ twins (3 %) is a strong indication of a genetic component [6]. Studies have identified candidate genes specifically involved in hind limb development [7, 8] and muscle contractile proteins [9].

Various environmental influences have been shown to increase the risk of clubfoot in that they may affect the developing foetus in different ways including via genetic alterations, deformation, or growth arrest. The incidence has been reported to rise with increased maternal alcohol consumption [10], smoking [11, 12], and if the mothers had undergone amniocentesis especially where a leak of amniotic fluid had occurred [13, 14]. In addition seasonal variations have been reported, with an increased incidence during the winter months and a proposed correlation to an increased maternal body temperature during the early phase of embryonic development [15] or infectious agents [16].

In animal studies a clubfoot-like deformity has been induced in different ways, and with the human studies in mind the most interesting are: the raise of body temperature of pregnant hamsters [17], irradiation of pregnant mice [18], and immobilisation by curarizing chick foetuses [19].

Despite previous studies, the aetiology of congenital clubfoot and the role of genetic and environmental factors remains unclear. The purpose of this study was to establish a congenital clubfoot twin cohort that enables us to estimate heritability, in order to determine to what extent genetic and/or environmental factors contribute to the aetiology of congenital clubfoot and to asses whether there was a change in the prevalence over time. Furthermore, we estimate the best fitting aetiological model for the phenotype.

## Materials and methods

The Odense-based Danish Twin Registry (DTR) is quite unique, as it contains data on all approximately 85,000 twin pairs born in Denmark over the last 140 years. It was the first population-based twin registry and one of the largest existing today [20]. All twins registered in DTR born from 1931 through 1982, who had previously consented to be contacted, received a 17-page questionnaire (twin omnibus) in the spring of 2002. For the birth cohorts 1931–1952, 70 % of all twin births have been ascertained. Based on a thorough search in selected parish registers, it has been estimated that almost 90 % of the eligible twin pairs were ascertained, taking into account infant mortality and survival to age 6 years. From 1968 there is complete ascertainment of twin pairs with both members live born, and since 1973 complete ascertainment of all multiple births due to the use of the Medical Birth Register [21].

The information on zygosity in the DTR is based on a validated method consisting of four questions of similarity with an accuracy of 95 % [22].

The prevalence of self-reported congenital clubfoot was calculated using the total number of answers as the general population and stratified according to gender.

Heritability is defined as the proportion of the total phenotypic variance that is attributable to genetic variance. Heritability was estimated in a generalized structural equations model, which combines a structural part with a liability threshold part. A liability threshold model assumes that the dichotomous distribution of clubfoot (affected versus non-affected) reflects an underlying normally distributed liability in the population. When a threshold value is exceeded, an individual is affected; otherwise not. Different thresholds in different groups, e.g. the two sexes or siblings versus unrelated individuals, reflect different loads of genetic and environmental risk factors in the groups, and thereby also the prevalence of the trait. Adjustment for age and sex effects on clubfoot was done through a probit regression model on the thresholds. These are standard assumptions in quantitative genetic analysis of categorical traits [23].

The structural equation model quantifies sources of individual variation by decomposing the total phenotypic variance into genetic and environmental variances [24]. The genetic variance component can be further divided into an additive (A) genetic component (representing the influence of alleles at several loci acting in an additive manner) and a non-additive (D) genetic component (representing intra- and inter-locus interaction). The environmental variance component is subdivided into a common (C) environmental component (representing environmental factors affecting both twins in a pair, and a source of similarity) and an individual/unique environmental component (E) (environmental factors acting differently in the twins of a pair and making them dissimilar). E also contains measurement error.

Tetrachoric correlation is a special case of the polychoric correlation, applicable when the observed variables are dichotomous. The tetrachoric correlations are used to decide which etiological model to fit. Selection of the best-fitting submodel is based on a balance between goodness of fit and parsimony [24]. The Akaike information criterion (AIC) is a measure of the goodness of fit of a statistical model [25]. AIC describes the balance between accuracy and complexity of the models, and therefore provides a means for model selection based on the lowest AIC value. Parsimony is a non-parametric statistical method where the evolutionary model that has the highest probability of producing the observed data is the most likely model. Estimation of tetrachoric correlations, heritability, and the best-fitting etiological model were done using the MX software [24, 26]. We also used SPSS software version 6 and Epi-Info. All the scientific-ethical committees of Denmark approved the study.

## Results

A total of 46,418 twins received and 34,944 (75 %) returned the questionnaire. 34,485 (99 % of the responders) answered the question ‘Were you born with clubfoot?’ The gender distribution in these was 15,731 (46 %) males and 18,754 (54 %) females.

Ninety-four answered ‘Yes’ to the above question, giving an overall self-reported prevalence of congenital clubfoot of 0.0027 (95 % c.i. 0.0022–0.0034), with male and female prevalences of 0.0031 (95 % c.i. 0.0022–0.0041) and 0.0025 (95 % c.i. 0.0018–0.0033), respectively (*p* = 0.29).

Fifty-five complete (both twins answered the question) twin pairs were identified, representing 12 monozygotic (MZ), 22 same-sex dizygotic (DZss), 18 opposite-sex dizygotic (DZos), and 3 pairs with unclassifiable zygosity. Only twins with a classifiable zygosity were used in the subsequent analysis.

Tetrachoric correlations were 0.81 (0.49–0.95) for MZ twins and 0.56 (0.25–0.77) for DZ twins; the difference in correlations indicates a possible genetic component. Variance component models ACE, ADE, and submodels were fitted to the data. Results are presented in Table 1. The ACE model was superior to the ADE model. Neither sub-model AE nor CE differed significantly from the larger ACE model, and when examining AIC, the CE model showed the best fit. However, due to the small statistical power, we hypothesise that the larger, more complex model ACE could be the better model.

**Table 1:** Results of structural equation modelling

	A	D	C	E	AIC	*p* values^a^	*p* values^b^
ACE	0.30 (0.00–0.93)		0.51 (0.00–0.86)	0.19 (0.05–0.48)	−29,027.88		
ADE	0.85 (0.09–0.96)	0.00 (0.00–0.76)		0.15 (0.04–0.39)	−29,025.59		
AE	0.85 (0.61–0.96)			0.15 (0.04–0.39)	−29,027.59	0.13	1.00
DE		0.84 (0.58–0.96)		0.16 (0.04–0.42)	−29,023.19		0.01
CE			0.72 (0.50–0.87)	0.28 (0.13–0.50)	−29,029.21	0.41	
E				1.00 (1.00–1.00)	−29,002.13	0.00	0.00

Selection of the best-fitting sub-model is based on the lowest AIC value and corresponding *p* values. The lines are the sub-models with different combinations of genetic and environmental contributions as described above. The rows are the quantification of the different component's contribution within the sub-models.

^a^Comparing sub-model to ACE model

^b^Comparing sub-model to ADE model

Choosing the ACE model, we found a heritability of clubfoot of 30 %.

Regression coefficient for age was −0.002 (−0.011 to 0.005), indicating that there has been no change in prevalence of clubfoot over the 50-year age span we examined.

## Discussion

The present study is based on one of the largest twin cohorts in the world. In the study we propose that the best model was the ACE, suggesting both a genetic and an environmental component in congenital clubfoot.

We calculated a regression coefficient of age. It tells us whether there is a tendency for the diagnosis to become more or less frequent over time. Since it is very close to 0 (−0.002), we believe that there has been no change in prevalence of clubfoot over the 50-year age span we examined.

Twin studies are accepted as unique in discriminating the contribution of genetic and environmental factors in the phenotypic variance. This discrimination is purest in a congenital disease, since any influence of differences in the postnatal environment is avoided. It is important that the twin cohort is population-based, since compared to population-based studies the disease-based studies have a tendency toward overrepresentation of MZ and concordant pairs [27, 28]. However, it should be kept in mind that since the zygosity was determined by questionnaire, this might affect the identification of zygosity, but the frequency of misclassification was no higher than 5 % [29].

The cardinal challenge in questionnaire-based studies is the identification of the patients. In the present study on congenital clubfoot we consider this to be a reliable method, since this distinct disorder is not easily overlooked or mistaken for other frequent disorders. However, some patients may have other foot deformities such as metatarsus varus, pes cavus, etc. The treatment of clubfoot in these cohorts was splinting and/or operation. The final results of the treatment were inferior to the present-day Ponseti technique. We do not expect patients to be unaware of having had clubfoot, however well corrected. Less than 50 % of clubfeet will be corrected in one operation, around 80 % in two operations, and approximately 90 % after three operations [30]. Long-term follow-up after surgical correction indicates that many patients suffer from pain, limping, and overall reduced foot function [31].

The prevalence of 0.27 % is higher than the 0.1–0.2 % reported for other Scandinavians. This might be due to an over-estimation in the self-reported “diagnosis”. If not, we must conclude that in this large and comprehensive twin cohort, there is an increased risk of clubfoot [32].

Previous studies have demonstrated that genetic factors might play a significant role; we found evidence of an environmental component and a probable genetic component in the aetiology of congenital clubfoot. Because of the incidence of congenital clubfoot and despite the size of this big twin cohort, the number of twins with clubfoot only yielded a relatively small amount of data. Due to this and the very similar AIC, we have chosen the largest model to explain heritability of clubfoot. Based on the findings presented here, we conclude that environmental factors must play a significant role in the aetiology of congenital clubfoot. And there is a possible genetic factor as well and a heritability of 30 %. Our finding lends no strong support for the theory of a major genetic factor in clubfoot.

To clarify the role of inheritance in congenital clubfoot, further studies are necessary, and the focus should be on interacting factors, thus rendering a certain genetic constitution more or less susceptible to environmental influences and epigenetic changes.
